# Defining internal target volume using positron emission tomography for radiation therapy planning of moving lung tumors

**DOI:** 10.1120/jacmp.v15i1.4600

**Published:** 2014-01-06

**Authors:** Adam C. Riegel, M. Kara Bucci, Osama R. Mawlawi, Moiz Ahmad, Dershan Luo, Adam Chandler, Tinsu Pan

**Affiliations:** ^1^ Department of Radiation Medicine North Shore LIJ Health System New Hyde Park NY; ^2^ Department of Radiation Oncology University of Texas M. D. Anderson Cancer Center Houston TX; ^3^ Department of Imaging Physics University of Texas M. D. Anderson Cancer Center Houston TX; ^4^ Department of Radiation Oncology Stanford University School of Medicine Stanford CA; ^5^ Department of Radiation Physics University of Texas M. D. Anderson Cancer Center Houston TX; ^6^ GE Healthcare Waukesha WI USA

**Keywords:** PET/CT, treatment planning, tumor delineation, lung cancer

## Abstract

Substantial disagreement exists over appropriate PET segmentation techniques for non‐small cell lung cancer. Currently, no segmentation algorithm explicitly considers tumor motion in determining tumor borders. We developed an automatic PET segmentation model as a function of target volume, motion extent, and source‐to‐background ratio (the VMSBR model). The purpose of this work was to apply the VMSBR model and six other segmentation algorithms to a sample of lung tumors. PET and 4D CT were performed in the same imaging session for 23 patients (24 tumors) for radiation therapy planning. Internal target volumes (ITVs) were autosegmented on maximum intensity projection (MIP) of cine CT. ITVs were delineated on PET using the following methods: 15%, 35%, and 42% of maximum activity concentration, standardized uptake value (SUV) of 2.5 g/mL, 15% of mean activity concentration plus background, a linear function of mean SUV, and the VMSBR model. Predicted threshold values from each method were compared to measured optimal threshold values, and resulting volume magnitudes were compared to cine‐CT‐derived ITV Correlation between predicted and measured threshold values ranged from slopes of 0.29 for the simplest single‐threshold techniques to 0.90 for the VMSBR technique. $R^{2}$ values ranged from 0.07 for the simplest single‐threshold techniques to 0.86 for the VMSBR technique. The VMSBR segmentation technique that included volume, motion, and source‐to‐background ratio, produced accurate ITVs in patients when compared with cine‐CT‐derived ITV.

PACS number: 87.57.nm

## I. INTRODUCTION

The use of fluorodeoxyglucose positron emission tomography (FDG‐PET) in radiation therapy planning of lung cancer grew substantially after the hardware fusion of PET/CT scanners in the last decade facilitated the inherent coregistration of anatomical and functional imaging. The ability of PET/CT to image distant metastases can change treatment intent from curative to palliative,^1^, ^2^, ^3^, ^4^ and the use of PET/CT for target definition can significantly change gross tumor volumes (GTVs) by distinguishing between malignant tissue and atelectasis.^5^, ^6^, ^7^, ^8^

A noted advantage of PET/CT for target delineation is the reduction of interobserver variation.^2^, ^4^, ^5^, ^9^ If a target delineation method is not clearly defined, however, significant differences between observers may remain.^10^, ^11^ Automated segmentation techniques can decrease interobserver variation, and several methods based on phantom and/or patient imaging have been investigated,^6^, ^12^, ^13^, ^14^, ^15^, ^16^, ^17^, ^18^, ^19^ Erdi et al.^(16)^ found that 36%‐44% of maximum activity concentration (${\text{AC}}_{\text{max}}$) correlated well with known sphere volumes in a stationary phantom and, in a later publication, the group settled on a single threshold of 42%.^(6)^ Paulino and Johnstone^(17)^ suggested a standardized uptake value (SUV) threshold of 2.5 g/mL, a value that originated from differentiation of benign versus malignant lesions in diagnostic PET imaging of non‐small cell lung cancer.^(20)^ Nestle et al.^(18)^ proposed thresholds at 15% of mean activity concentration (${\text{AC}}_{\text{mean}}$) plus background activity concentration to compare with CT‐defined GTV Black et al.^13^ developed a linear function of mean SUV using CT scans of different‐sized spheres.

Few studies actually consider motion in PET‐defined target volumes, despite the trend towards 4D imaging and increased margins to treat the “motion envelope” of the tumor. Acknowledging that moving objects are blurred in PET due to long acquisition times, Caldwell et al.^(15)^ found that a threshold of 15% of ${\text{AC}}_{\text{max}}$ can incorporate motion extent into the resulting contour. In a phantom study, Okubo et al.^(19)^ found that 35% was the optimal threshold for large stationary or moving spheres.

We developed a segmentation method which incorporated tumor size, motion extent, and source‐to‐background ratio (SBR) in the determination of optimal activity concentration threshold.^(21)^ Using an extensive series of phantom scans at varying sphere volume, sinusoidal motion extent, and SBR, multiple regression techniques were used to fit an analytical function called volume/motion/SBR or “VMSBR” model. This model described the threshold which best matched the motion envelope of the tumor as defined on cine CT.

The purpose of this study was to apply the VMSBR model and six other published segmentation methods to a group of lung tumors previously planned for radiation therapy. Ultimately, we hope to apply the VMSBR model to complex tumors where CT and PET can be used in a complementary fashion.

## II. Materials and Methods

### A. Imaging

Lung cancer patients who underwent 4D CT and PET/CT simulation in the same imaging session and demonstrated one or more solid lesions with relatively homogeneous uptake on PET without invasion into the chest wall or mediastinal regions were retrospectively included in the study under an IRB‐approved protocol. PET/CT and 4D CT simulations were performed on a Discovery ST 8‐slice PET/CT scanner (GEMS, Waukesha, WI). The 4D CT protocol used 120 kV, 100 mA, 0.5 s gantry rotation, 2 cm axial beam width, 2.5 mm slice thickness, 0.25 s cine interval, and cine duration equal to 1 average breathing cycle plus 1 s. For PET imaging, patients were injected with 477 to 740 MBq of ${}^{18}F‐\text{FDG}$ and PET was acquired in 2‐D mode for 3 min per bed position from base of skull to midthigh. Attenuation correction was performed with respiratory‐averaged CT to ensure registered CT and PET data.(22) Images were reconstructed with OSEM iterative reconstruction utilizing 30 subsets and two iterations. Both PET and CT imaging used a 50 cm field of view with a 128×128 and 512×512 image matrix respectively. PET slice thickness was 3.27 mm and CT slice thickness was 2.5 mm. PET and 4D CT images were transferred to Pinnacle3 version 8.1w (Philips Medical Systems, Madison, WI) for contouring.

## B. Target delineation on CT and PET

To form ITV on $\text{CT}({\text{ITV}}_{\text{CT}})$, gross tumor was contoured on the maximum intensity projection (MIP) generated from cine‐CT images.^23^, ^24^ Cine‐CT images are unsorted reconstructed CT images that are retrospectively sorted to form 4D CT phase imaging in General Electric CT scanners (GE Healthcare, Waukesha, WI). Since forming MIP from cine CT includes all images during a respiratory cycle (not a sampled subset), these MIPs can often more accurately capture full motion extent.^(23)^ ITVs were generated using seed‐based 3D region growing autosegmentation, which was used to minimize observer variation and bias. A threshold of ‐425 HU was used to limit the region‐growing algorithm.^25^, ^26^ A radiation oncologist reviewed the cine‐generated MIP and 4D CT phase images and adjusted the ${\text{ITV}}_{\text{CT}}$ contours if necessary.

It should be noted that ITV in this work refers to the motion envelope of the tumor — that is, the voxels that contain tumor at some point during the respiratory cycle. This concept differs from the ICRU‐defined ITV, which includes a margin for microscopic extension and a population‐based expansion for motion.^(27)^ Here, we explicitly determine the motion envelope of the tumor with cine CT.

ITVs were delineated on PET using six segmentation methods from the literature (collectively referred to as ${\text{ITV}}_{\text{PET}}$, summarized in Table 1). ${\text{ITV}}_{15\%},{\text{ITV}}_{35\%},{\text{ITV}}_{42\%}$, and ${\text{ITV}}_{2.5}$ were all single thresholds of ${\text{AC}}_{\text{max}}$ or standardized uptake value (SUV).^6^, ^15^, ^16^, ^17^, ^19^ ${\text{ITV}}_{15\%+\text{BG}}$ was formed by calculating 15% of ${\text{AC}}_{\text{mean}}$ and adding the result to a background measurement. As described in Nestle et al.,^(18)^ ${\text{AC}}_{\text{mean}}$ was measured in pixels above 70% of ${\text{AC}}_{\text{max}}$ and background was measured in a small region of interest (ROI) defined in the adjacent anatomical structure with the highest background activity. For ${\text{ITV}}_{\text{SUVmean}}$, a starting threshold was required to measure ${\text{SUV}}_{\text{mean}}$ and was not explicitly stated. As per the authors’ suggestion, we chose an arbitrary threshold ($70\%\;{\text{AC}}_{\text{max}}$) and iterated through the regression function five times.^(13)^ All ITVs were formed using seed‐based, region‐growing automatic segmentation in Pinnacle^3^.

**Table 1:** Tumor delineation methods

*Delineation Technique*	*Notation*
15% of ${\text{AC}}_{\text{max}}$ ^(15)^	${\text{ITV}}_{15\%}$
35% of ${\text{AC}}_{\text{max}}$ ^(19)^	${\text{ITV}}_{35\%}$
42% of ${\text{AC}}_{\text{max}}$ ^6^, ^16^	
${\text{ITV}}_{42\%}$
SUV=2.5g/mL ^(17)^	${\text{ITV}}_{25}$
15% of ${\text{AC}}_{\text{mem}+\text{BG}}$ ^(18)^	${\text{ITV}}_{15\%+\text{BG}}$
$0.307\times {\text{SUV}}_{\text{mean}}+0.588$ ^(13)^	${\text{ITV}}_{\text{SUVmean}}$
volume/motion/SBR^(21)^	${\text{ITV}}_{\text{VMSBR}}$
${\text{AC}}_{\text{max}}=\text{maximum activity concentration}$; ${\text{AC}}_{\text{mean}}=\text{mean activity concentration}$; SUV=standardized uptake value; BG=background; ITV=internal target volume.

### C. New segmentation method of volume, motion, and SBR

**Figure 1:**
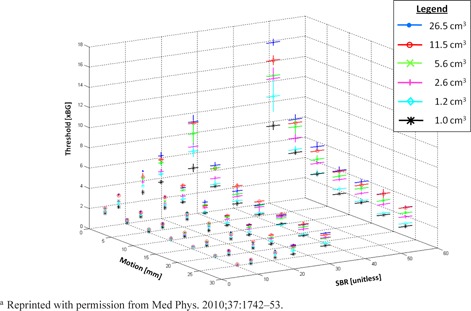
Phantom‐derived optimal activity concentration thresholds versus motion, source to background, and volume (denoted by different symbols). Threshold values are normalized to background. These values were used to generate the analytical expression in Eq. (1).^a^

The VMSBR model was developed by scanning a NEMA IEC phantom containing six spheres of varying diameter (10‐37 mm) at varying sinusoidal motion amplitudes (0‐30 mm) and varying SBR (0‐50) for a total of 252 combinations of these parameters.^(21)^ Optimal thresholds as percent of ${\text{AC}}_{\text{max}}$ were geometrically fit to the motion envelopes of the moving spheres by minimizing the surface separation between the two volumes. Results of these phantom analyses are shown in Fig. 1. An analytical function was fit to the optimal thresholds and the resulting function is shown in Eq. (1), where *w* is optimal threshold normalized to background, x is tumor volume in cubic centimeters, *y* is motion extent in millimeters, and *z* is SBR (which is unitless). The model was initially validated in our previous work using a phantom dataset acquired on a different PET/CT scanner with different reconstruction parameters (the same scanner and reconstruction parameters used in the current study) and three moving lung tumors. Surface agreement between PET and reference CT volumes was less than 2 mm.^(21)^

(1) ln(w)=0.0634x13+0.1202y13+0.7327(ln(z))+0.0597(x13ln(z))−                 0.1221(y13ln(z))−0.0248(xy)13−0.9504

To determine input for Eq. (1), tumor volume and motion were determined by autosegmenting the end‐inspiration and end‐expiration phases of 4D CT. Volume was measured at end‐expiration, and motion was measured as the distance between the end‐inspiration and end‐expiration centroids. SBR of the PET image was determined by dividing tumor ${\text{AC}}_{\text{mean}}$ by background ${\text{AC}}_{\text{mean}}$. ${\text{AC}}_{\text{mean}}$ in the tumor was determined by creating a ROI at 80% of ${\text{AC}}_{\text{mean}}$ and measuring the ${\text{AC}}_{\text{mean}}$ in the ROI. Background was measured by segmenting the ipsilateral lung, removing any areas of high uptake (e.g., tumors, inflammation, imperfect segmentation near the mediastinum), and measuring ${\text{AC}}_{\text{mean}}$.^(28)^ If a lesion was located close to the chest wall or mediastinum and the contour “bled” into these areas, ${\text{ITV}}_{\text{PET}}$ was manually trimmed based on structural boundary information from CT to eliminate spillover.

The measured SBR value was degraded by partial volume averaging^(29)^ and tumor motion.^(30)^ When Eq. (1) was derived from phantom scans, the nondegraded SBR value was used for regression. We therefore multiplied the degraded SBR in patient scans by a recovery coefficient (RC) derived from phantom scans to correct for size and motion degradation (Eq. (2)), where x is volume in cubic centimeters and *y* is motion in millimeters.^(21)^

(2) $$RC=0.1991x^{-1}+0.0136y+0.0725x^{-1}y+0.8839$$

The recovered SBR value, tumor volume, and motion were plugged into Eq. (1) to generate the optimal threshold for each lesion. ITV produced using the VMSBR regression model (${\text{ITV}}_{\text{VMSBR}}$) was created by seed‐based, region‐growing autosegmentation of the PET image.

### D. Analysis

**Figure 2:**
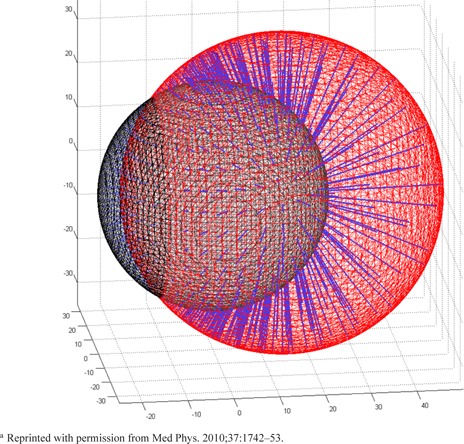
Two simple spherical meshes are compared via the surface separation algorithm. From each sampling point on the “reference” mesh (in this case, the smaller sphere), the closest point on the other mesh surface is calculated. This

Accuracy of the segmentation techniques was assessed two ways. First, the threshold value generated by each technique was compared to the optimal threshold value. The optimal threshold value was measured via the surface separation algorithm in a manner similar to our previous work on phantoms.^(21)^ ROIs were segmented on PET images in 1% intervals of ${\text{AC}}_{\text{max}}$, converted to triangular mesh surfaces, and compared with ${\text{ITV}}_{\text{CT}}$ (Fig. 2). The volume producing the smallest surface separation was deemed “optimal”. Predicted threshold values from each segmentation technique were correlated with this measured optimal threshold. The second analysis compared ${\text{ITV}}_{\text{PET}}$ volume magnitudes with ${\text{ITV}}_{\text{CT}}$ using paired *t*‐tests method was used to determine the optimal activity concentration threshold which best matched ${\text{ITV}}_{\text{CT}}$ for each patient.

Correlations between measured optimal values and predicted values are shown in Fig. 5.^a^

## III. RESULTS

Twenty‐four tumors (23 patients) fit our criteria, were registered, and were included in the analysis. ${\text{ITV}}_{15\%}$ could not be segmented for six tumors because 15% of ${\text{AC}}_{\text{max}}$ fell below the background activity concentration. Similarly, ${\text{ITV}}_{2.5}$ could not be segmented for two tumors for the same reason. As such, *t*‐tests were performed with paired values, limiting the sample to 18 and 22 tumors respectively.

**Figure 3:**
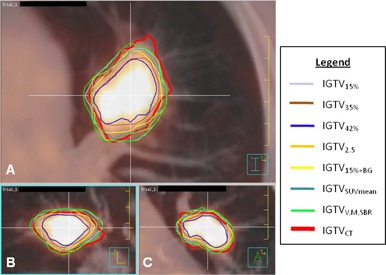
PET/CT images of tumor 1 with internal target volume (ITV) contours: (a) transverse, (b) sagittal, and (c) coronal. ${\text{ITV}}_{\text{CT}}$, shown in red, was delineated on the maximum intensity projection. Other ITVs were delineated on PET using methods described in Table 1. The VMSBR model (${\text{ITV}}_{\text{VMSBR}}$) is shown in green.

**Figure 4:**
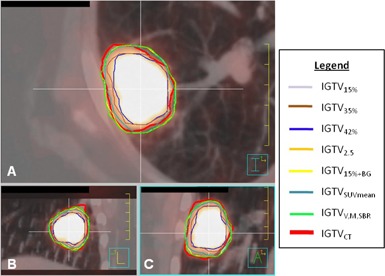
PET/CT images of tumor 13 with internal target volume (ITV) contours: (a) transverse, (b) sagittal, and (c) coronal. ${\text{ITV}}_{\text{CT}}$, shown in red, was delineated on the maximum intensity projection. Other ITVs were delineated on PET using methods described in Table 1. The VMSBR model (${\text{ITV}}_{\text{VMSBR}}$) is shown in green.

Figures 3 and 4 illustrate typical patient examples for all segmentation methods. ${\text{ITV}}_{\text{CT}}$ is denoted by the thick red line.

**Figure 5:**
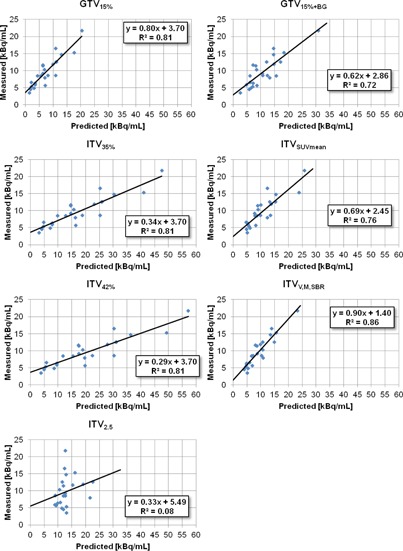
Correlation between predicted threshold values and measured optimal threshold values for each segmentation method. Best‐fit lines, their equations, and $R^{2}$ values are shown in each plot.

Volume, motion, “source”, and background measurements for the 24 tumors are shown in Table 2. Linear correlations (y=mx+b) of the measured thresholds and the predicted thresholds for each segmentation technique are shown in Fig. 5. The correlation of ${\text{ITV}}_{\text{VMSBR}}$ thresholds with measured values has a slope close to 1 (m=0.90), a y‐intercept closest to 0 (b=1.40), and the highest $R^{2}$ value ($R^{2}=0.86$).

Volume magnitude differences are summarized in Table 3. ${\text{ITV}}_{\text{SUVmean}},{\text{ITV}}_{15\%+\text{BG}}$, and ${\text{ITV}}_{\text{VMSBR}}$ all yielded nonsignificant differences with ${\text{ITV}}_{\text{CT}}$, with the VMSBR technique yielding the lowest percent difference (−5.15%). All single‐threshold techniques yielded volumes whose differences with ${\text{ITV}}_{\text{CT}}$ were statistically significant.

**Table 2:** Volume, motion, tumor, and background characteristics of 24 lung tumors

*Lesion*	*Location*	*Volume (cm^3^)*	*Motion (mm)*	*Tumor AC (Bq/mL)*	*Background AC A (Bq/mL)*
1	RLL	21.1	10.8	99801.6	3576.5
2	RUL	15.3	7.8	63799.8	2515.8
3	RLL	1.1	10.3	11910.4	3127.5
4	RLL	0.8	6.2	60414.2	2267.4
5	LUL	1.0	6.6	67181.7	5692.0
6	RUL	28.0	1.5	38936.3	2890.4
7	RLL	2.5	13.6	33286.9	2781.2
8	LUL	1.5	2.4	79330.4	3702.4
9	RUL	0.7	4.6	36930.2	3118.9
10	RML	0.6	2.2	27270.3	3110.9
11	RUL	13.6	1.0	121006	3043.0
12	LUL	1.1	8.0	10314.4	3202.8
13	LUL	10.3	8.6	46834.6	1953.3
14	LLL	0.4	8.8	12710.3	3078.1
15	RUL	3.6	4.0	20158.7	2750.5
16	LLL	2.2	15.0	18006.2	3510.5
17	LUL	4.8	3.1	21106.1	2330.7
18	RUL	2.7	2.7	42787.6	1809.4
19	RLL	0.7	1.5	38805.8	2488.2
20	RUL	1.6	6.3	65668.8	3080.7
21	LUL	1.5	2.7	41978.3	3384.9
22	LUL	5.2	0.6	65254.3	3680.7
23	LUL	0.1	1.0	8586.72	2409.0
24	LUL	0.5	5.1	42631.4	3683.0

AC=activity concentration; RUL=right upper lobe; RML=right middle lobe; RLL=right lower lobe; LUL=left upper lobe; LLL=left lower lobe.

**Table 3:** Comparison of ${\text{ITV}}_{\text{PET}}$ with ${\text{ITV}}_{\text{CT}}$ for different segmentation methods

*Notation*	$\text{Volume}\pm \text{SEM}({\text{cm}}^{3})$	*p‐value*	$({\text{ITV}}_{\text{PET}}-{\text{ITV}}_{\text{CT}})\pm \text{SEM}$	*Percent Difference (%)*
${\text{ITV}}_{15\%}$ ^a^	10.93±2.71	0.01^c^	+1.05±0.89	±10.7%
${\text{ITV}}_{35\%}$	4.15±0.99	<0.01 ^c^	−4.17±1.24	−50.1%
${\text{ITV}}_{42\%}$	3.12±3.93	<0.01 ^c^	−5.21±1.38	−62.5%
${\text{ITV}}_{2.5}$ ^b^	6.93±2.14	<0.01 ^c^	−1.99±0.73	−22.3%
${\text{ITV}}_{2.5}$	6.36±1.67	0.14	−1.96±0.60	−23.6%
${\text{ITV}}_{\text{SUVmean}}$	7.02±1.67	0.33	−1.30±0.65	−15.7%
${\text{ITV}}_{\text{VMSBR}}$	7.89±1.76	0.39	−0.43±0.55	−5.15%
${\text{ITV}}_{\text{CT}}$	8.32±2.16	—	—	—

a ^a^ N=18. Statistics calculated using paired values.

b ^b^ N=22.

c ^c^ Statistically significant differences.

SEM=standard error of the mean.

## IV. DISCUSSION

Definition of target volumes using PET has been the topic of much research in recent years, but little agreement exists on how to use PET to define a target volume.^31^, ^32^, ^33^ The current study compares seven contouring methods of varying complexity, including a new PET segmentation method developed using a series of phantom calibrations that incorporates tumor volume, motion, and SBR into a threshold‐producing analytical function.

The methodology utilized in the current study of comparing PET‐segmented volumes to CT‐segmented volumes is similar to several publications in the literature.^18^, ^34^, ^35^, ^36^ Hanna et al.^(34)^ compared volumes from three single‐threshold and two SBR‐based PET segmentation techniques to 4D CT‐derived ITV and found that no segmentation algorithm adequately matched. Schaefer et al.^(35)^ used regression methods to develop an SBR‐based approach and validated the technique on eight NSCLC patients by comparing PET volumes to CT volumes delineated by a radiation oncologist. The investigators found that percent volume difference ranged from ‐9% to +28%, averaging 6% larger in PET than CT. The authors attributed this positive skew to respiratory motion. Lamb et al.^(36)^ used 4D PET to create “PET‐MIP” image sets and segmented PET ITVs at thresholds ranging from 20% to 40% of maximum SUV. The investigators compared these volumes to ITVs drawn by radiation oncologists on the 4D CT MIP and found good overlap between PET‐MIP volumes and CT‐MIP volumes.

The correlation of ${\text{ITV}}_{\text{VMSBR}}$ threshold and optimal threshold produced a slope close to 1(m=0.90), implying good correlation of predicted and optimal values, the y‐intercept closest to 0(b=1.40), implying little systematic over‐ or underestimation, and the highest $R^{2}$ value ($R^{2}=0.86$), implying the tightest spread of predicted values.

Previously, we found that SBR was the most influential variable in threshold prediction.^(21)^ This result was consistent with Brambilla et al.^(14)^, who found that SBR was more influential than size for spheres over 10 mm in diameter. The fact that both ${\text{ITV}}_{15\%+\text{BG}}$ and ${\text{ITV}}_{\text{SUVmean}}$ explicitly or implicitly account for SBR and both perform nearly as well as our technique suggests that considering SBR in segmentation is critical. Other authors have previously put forth this notion.^18^, ^28^, ^33^ Our results indicate that considering motion, in addition to size and SBR, can further increase the accuracy of PET delineation when compared with 4D CT or cine CT.

The current manifestation of the VMSBR model represents a first approximation to many moving tumors and there are several ways the model can be improved. The measurement of SBR, and particularly the measurement of background, is evidently critical for segmentation and factors that affect SBR should be further investigated. Additionally, several assumptions were made in the development of the VMSBR model that should be explored further. First, the model was developed using one‐dimensional sinusoidal motion, which is obviously inaccurate for many lung tumors.^37^, ^38^ Second, the model was developed with spherical objects; tumors with spiculations or substantial asymmetry may not conform to the model. Third, we assumed that motion during 4D CT acquisition was essentially the same as motion during PET acquisition. Motion patterns, including amplitude, frequency, and baseline position, can change over time^(39)^, leading to mismatching volumes or misregistration. Fourth, the model was developed assuming homogeneous uptake, which is a reasonable assumption for smaller tumors, but not for larger tumors where heterogeneity, hypoxia, or necrosis occur. The impact of AC heterogeneity on the VMSBR model should be evaluated.

Nevertheless, the VMSBR model, along with other SBR‐inclusive algorithms, produced promising results. Single‐threshold techniques may be simpler, but Biehl et al.^(12)^ suggest that single thresholds are inappropriate for target delineation and, given the results of this work, we must concur. Nestle et al.^(18)^ found that 40% of ${\text{AC}}_{\text{max}}$ underestimates CT volumes with a population‐based motion expansion. We had similar findings for both ${\text{ITV}}_{42\%}(-62.5\%)$ and ${\text{ITV}}_{35\%}(-50.1\%)$ when compared with a motion envelope explicitly determined on cine CT. Though access to 4D CT for motion measurement may be a limitation, considerable motion information can be extracted from image sets processed directly from cine CT.^23^, ^24^

The focus of the current study was segmentation of relatively homogeneous and spherical primary tumors where extent was easy to determine with CT. We wanted first to validate our model in simple clinical cases where PET and CT data are supplementary (they indicate the same volume), before applying the model to the larger lung cancer population where PET and CT are complementary (PET may indicate malignancies that are indiscernible on CT).^(40)^ In future work, we will assess the efficacy of the VMSBR model in complex primary tumors and mediastinal lymph nodes (which can demonstrate significant motion^41^, ^42^ for delineation of locally advanced non‐small cell lung cancer.

## V. CONCLUSIONS

A segmentation model for moving lung lesions in PET that incorporates tumor volume, motion, and SBR into determination of optimal activity concentration threshold was developed. The model, calibrated with an extensive series of phantom scans, was applied to 24 lung lesions to form ITVs. These ITVs, as well as six others generated using methods published in the literature, were compared with cine‐CT‐defined ITV. The VMSBR model produced ITVs that correlated well with ITVs generated from cine CT. Further research is required to examine portability of the model to different patient and scan conditions.

## Supporting information

Supplementary Material
