# Supplementary material for: Defining internal target volume using positron emission tomography for radiation therapy planning of moving lung tumors
Source: J Appl Clin Med Phys. 2014 Jan 6;15(1):279–89. doi: 10.1120/jacmp.v15i1.4600 (PMC5711243; doi:10.1120/jacmp.v15i1.4600)
Supplement: Supplementary file 1 — Supplementary Material [file ACM2-15-279-s001.pdf]

American Association of Physicists in Medicine
One Physics Ellipse
College Park, MD 20740-3846
(301) 209-3350
Fax (301) 209-0862
http://www.aapm.org

Office of the Executive Director
Angela R. Keyser
Phone: 301-209-3385 Fax: 301-209-0862
E-mail: akeyser@aapm.org

DATE OF REQUEST: 5/9/2013

FROM:

Adam C. Riegel, Ph.D.
Dept. of Radiation Medicine, North Shore LIJ Health System, 270-05 76th Ave, New Hyde Park, NY 11040

EMAIL ADDRESS: ariegel@nshs.edu

1. **Permission is granted to:**

   Adam C. Riegel, Ph.D.
   Dept. of Radiation Medicine, North Shore LIJ Health System, 270-05 76th Ave, New Hyde Park, NY 11040

2. **Permission is requested to use the following material:**

   Riegel AC, Bucci MK, Mawlawi OR, Johnson V, Ahmad M, Sun X, Luo D, Chandler AG, Pan T, "Target definition of moving lung tumors in positron emission tomography: Correlation of optimal activity concentration thresholds with object six, motion extent, and source-to-background ratio." Med. Phys. 2010 Apr;37(4):1742-52, Figures 3 and 5.

3. **For what purpose:**

   I would like to use these two figures in an upcoming paper. Thank you.

Authors seeking permission must also notify the first author of the article from which permission is being sought.

Permission is hereby granted: Angela R. Keyser 05/14/13

Signature **Date**
